# Carbon Monoxide-Releasing Molecule-3 Suppresses Tumor Necrosis Factor-*α*- and Interleukin-1*β*-Induced Expression of Junctional Molecules on Human Gingival Fibroblasts via the Heme Oxygenase-1 Pathway

**DOI:** 10.1155/2020/6302391

**Published:** 2020-04-28

**Authors:** Jia Lv, Yongsheng Liu, Shuhan Jia, Yuna Zhang, Haoyang Tian, Jingyuan Li, Hui Song

**Affiliations:** ^1^Department of VIP Center, School and Hospital of Stomatology, Shandong University & Shandong Key Laboratory of Oral Tissue Regeneration & Shandong Engineering Laboratory for Dental Materials and Oral Tissue Regeneration, No. 44-1 Wenhua Road West, 250012 Jinan, Shandong, China; ^2^Department of Prosthodontics, Qilu Hospital of Shandong University, Jinan, Shandong, China

## Abstract

Human gingival fibroblast barrier dysfunction caused by inflammation contributes to gingivitis and can lead to inflammatory periodontal disease. The disease features include upregulated epithelial permeability, increased inflammatory mediators, and downregulated junctional complex molecules. Carbon monoxide- (CO-) releasing molecule-3 (CORM-3) is a water-soluble compound that has demonstrated anti-inflammatory effects in *in vitro* and *in vivo* studies. In this study, we aimed to investigate the effects of CORM-3 on the expression of tight and adherens junction molecules on human gingival fibroblasts (HGFs) stimulated with tumor necrosis factor-*α* (TNF-*α*) and interleukin-1*β* (IL-1*β*). HGFs were cultured from the explants of normal human gingival tissues, which were stimulated in the presence or absence of CORM-3. Epithelial barrier function was evaluated by paracellular permeability and junctional complex molecule expression analyses. The protein and mRNA expression levels of adherens junction molecules (VE-cadherin and *β*-catenin) and tight junction molecules (zona occludens-1, ZO-1) were studied using western blot analysis and reverse transcription-quantitative polymerase chain reaction (RT-PCR). The mRNA and protein expression levels of these cytokines were also analyzed in HGFs transiently transfected with HO-1 small interfering RNA (siRNA) in response to TNF-*α* and IL-1*β* stimulation. CORM-3 reduced permeability and enhanced the expression of junctional complex molecules (ZO-1, VE-cadherin, and *β*-catenin) in TNF-*α*- and IL-1*β*-induced HGFs. However, these effects of CORM-3 were attenuated when HO-1 siRNA was transiently transfected in HGFs. These findings indicate that CORM-3 exerts anti-inflammatory effects on TNF-*α*- and IL-1*β*-stimulated HGFs via the HO-1 pathway, which suggests the promising potential of CORM-3 in the treatment of inflammatory periodontal disease.

## 1. Introduction

Periodontal disease is an inflammatory disease characterized by the presence of pathogenic bacteria in subgingival plaques, resulting in soft tissue destruction, alveolar bone resorption, and eventual tooth loss [1–3]. The most prominent feature of periodontitis is the accumulation of immunoactive cells, such as monocytes/macrophages and lymphocytes and their cytokines in extravascular periodontal connective tissues. In particular, it has been demonstrated that the proinflammatory cytokines tumor necrosis factor-*α* (TNF-*α*) and interleukin-1*β* (IL-1*β*) play an important role in periodontal tissue destruction [4–6]. It has also been reported that TNF-*α* and IL-1*β* enhance leukocyte adhesion by upregulating chemokines, increasing barrier permeability, and downregulating tight and adherens junction molecule expressions [7].

Human gingival fibroblasts (HGFs) are one of the most abundant cell types in periodontal connective tissue. HGFs function as support cells for periodontal tissues and produce inflammatory mediators in response to proinflammatory stimuli and pathogens [8]. The important role of periodontal connective tissue in maintaining periodontal tissue integrity has been well studied, as well as its role in regulating the local inflammatory response. Within the cell junctional complex, tight junctions are largely responsible for controlling paracellular transport, whereas adherens junctions are primarily responsible for cell-to-cell adhesion [9, 10].

As the rate-limiting enzyme in heme degradation, heme oxygenase-1 (HO-1) induction represents an essential event in cellular responses to proinflammation to maintain cellular homeostasis [11, 12]. HO-1, one of the most responsive of the known induced enzymes, has been proven to act as a cellular biosensor. High levels of HO-1 can be induced within a few hours by many stimulants, such as hemoglobin, cytokines, and endotoxins. The pharmacological or genetic modulation of HO-1 induces nuclear localization and inhibits cell migration, proliferation, and invasion [13]. HO-1 metabolizes and produces biliverdin, Fe^2+^, and carbon monoxide (CO) [14]. CO has been shown to play important roles in multicellular events; for example, CO can inhibit cell proliferation and apoptosis [15], suppress inflammation [16], and protect organs against ischemia/reperfusion injury [17, 18]. The effect of CO is mediated by HO-1 induction, guanylate cyclase activation, and p38 MAPK signaling pathway regulation [19].

Extensive studies have shown that CO-releasing molecules (CORMs), which can release CO in a controllable manner under physiological conditions, can increase heme oxygenase-1 (HO-1) expression in various animal models and cell types. CO-releasing molecules (CO-RMs) belong to two major classes: metal carbonyl complexes containing ruthenium, manganese, or molybdenum, which carry CO bound to the transition metal, and boranocarbonates that contain metalloid boron rather than transition metals. Among CORMs, CORM-1 and CORM-2 are lipophilic; they have to be dissolved in organic solvents such as dimethyl sulfoxide (DMSO). CORM-3 (tricarbonylchloro(glycinato)ruthenium(II)) is fully water-soluble and can rapidly liberate CO when dissolved in physiological solutions, which shows more promising potential in clinical treatment in the future [20]. By delivering and carrying CO in a controllable way, CORM-3 may exert important pharmacological activities [21].

A previous study by our research group found that CORM-3 inhibits the expression of adhesion molecules in HGFs stimulated with TNF-*α* and IL-1*β* [21]. Thus, the objective of our present study was to determine the effects of CORM-3 on HGF barrier function following exposure to the inflammatory cytokines TNF-*α* and IL-1*β* and to elucidate the mechanism underlying this effect of CORM-3.

## 2. Materials and Methods

### 2.1. Reagents

CORM-3, human recombinant TNF-*α*, and IL-1*β* were purchased from Sigma-Aldrich (St. Louis, MO, USA); Dulbecco's modified Eagle's medium (DMEM) was purchased from HyClone (GE Healthcare Life Sciences, Logan, UT, USA); fetal bovine serum (FBS) was purchased from Biological Industries (Kibbutz Beit-Haemek, Israel), and 100x penicillin-streptomycin solution was obtained from Beijing Solarbio Science and Technology Co. Western blotting antibodies for *β*-catenin (ab32572, rabbit anti-*β*-catenin monoclonal antibody), VE-cadherin (ab33168, rabbit anti-VE-cadherin polyclonal antibody), heme oxygenase-1 (ab68477, rabbit anti-heme oxygenase-1 monoclonal antibody), ZO-1 (ab96587, rabbit anti-ZO-1 polyclonal antibody), and beta-actin (ab8226, mouse anti-*β*-actin monoclonal antibody) were from Abcam (Cambridge, UK). A glyceraldehyde-3-phosphate dehydrogenase (GAPDH) antibody (10494-AP, rabbit anti-GAPDH polyclonal antibody) and the secondary antibodies horseradish peroxidase- (HRP-) conjugated affiniPure goat anti-rabbit IgG(H+L) (SA00001-2) and HRP-conjugated affiniPure goat anti-mouse IgG(H+L) (SA00001-1) were purchased from Proteintech Group, Inc. (Chicago, IL, USA). Primers for beta-catenin, VE-cadherin, and ZO-1 were purchased from BioSune (Shanghai, China). Polyvinylidene fluoride (PVDF) membranes were purchased from Millipore (Bedford, MA, USA). A bicinchoninic acid (BCA) protein determination kit (Cat no. 23225) was purchased from Millipore (Bedford, MA, USA), and the PrimeScript RT reagent Kit with gDNA Eraser was from TaKaRa (TaKara Bio Inc., Japan).

### 2.2. Cell Culture

HGFs were isolated from the explants of normal gingival tissues with an explant culture technique. Informed consent was previously obtained from the patients before surgery. The study was approved by the Ethics Committee of the School of Stomatology, Shandong University (Jinan, China). Tissues were washed by Dulbecco's modified Eagle's medium (DMEM, HyClone, Logan, UT, USA) with penicillin G (100 U/ml) and streptomycin (100 mg/ml). Then, they were cut into 1-3 mm^2^ explants with scissors and cultured in 25 cm^2^ culture bottles (Corning Inc., Corning, NY, USA) supplemented with 100 U/ml penicillin G, 100 mg/ml streptomycin, and 20% heat-inactivated FBS in humidified air with 5% CO_2_ at 37°C. The seeding density of the cells was 1 × 10^5^ cells/ml. The medium was changed every 2-3 days before a confluent cell monolayer was formed. When the cells reached 90% confluence after 5-7 days, they were detached with 0.025% trypsin and 0.05% EDTA and subcultured at a ratio of 1 : 2. HGFs from passages 3-5 were used in the subsequent experiments. HGFs were pretreated with 100, 200, and 400 *μ*M CORM-3 for 6 h, respectively, before the cells were induced with 10 ng/ml TNF-*α* and 2 ng/ml IL-1*β* for another 24 h, unless otherwise specified.

### 2.3. Cell Proliferation Assay

Cell Counting Kit-8 (CCK-8) assays were used to assess the toxicity of CORM-3 at different concentrations on HGFs. HGFs were seeded and cultured with control medium in 96-well plates at a density of 5000 cells/well. HGFs were divided into six categories: TNF-*α* (10 ng/ml) and IL-1*β* (2 ng/ml) with increasing concentrations of CORM-3 were added to the wells and cultured for 24 h at 37°C. Unstimulated cells were used as a control. CORM-3 must be prepared freshly before the experiment by being dissolved in medium. Then, the 10 *μ*l CCK-8 reagent was added to each well, and the cells were incubated for another 2 h at 37°C. Subsequently, the optical density (OD) absorbance at 450 nm was measured using a SPECTROstar Nano ultraviolet spectrophotometer (SPECTRO Analytical Instruments GmbH, Germany). The entire experiment was repeated in triplicate.

### 2.4. Measurement of Fluorescein Isothiocyanate- (FITC-) BSA Flux across HGF Monolayers

To determine the changes in permeability across HGF monolayers, HGFs were seeded at a density of 1.0 × 10^5^ cells/well on top of 24-well transwell chambers with 6.5 mM diameter polyester membrane inserts (0.4 *μ*M pore size, COSTAR, USA) and grown to confluence. HGFs were cultured in medium containing 1% FBS for 24 h until a well-formed monolayer was observed. HGFs were then incubated with TNF-*α* (10 ng/ml) and IL-1*β* (2 ng/ml) with increasing concentrations of CORM-3 for 24 h. Unstimulated cells were used as a control. At the end of stimulation, the treatment medium was carefully removed from each plate well, and FITC-BSA (10 mg/ml, Sigma, USA) and equimolar amounts of unlabeled BSA were added to the top and bottom chambers with phenol red-free DMEM for 2 h at 37°C in the dark. The medium from different chamber wells was then transferred to a blank 96-well opaque plate for fluorescence measurement. Fluorescence intensity was quantified at 494 nm excitation and 520 nm emission (SpectraMax i3X, Molecular Devices, USA).

### 2.5. Immunofluorescence Staining

HGFs were seeded as a monolayer on 2% poly-L-lysine-coated cover glasses and serum-starved for 24 h. The cells were pretreated with TNF-*α* (10 ng/ml) and IL-1*β* (2 ng/ml) for 24 h, with increasing concentrations of CORM-3. Unstimulated cells were used as a control. The cells were washed with phosphate-buffered saline (PBS), fixed with 4% formaldehyde for 15 min at 4°C, and permeabilized with 1% Triton X-100 for 20 min at 4°C. HGFs were blocked with 5% BSA for 1 h and then exposed to the primary antibodies for VE-cadherin (1 : 250), ZO-1 (1 : 250), and beta-catenin (1 : 250) overnight at 4°C. The cover glasses were washed with PBS three times and then incubated with the appropriate secondary antibodies for 1 h at 37°C. After washing with PBS, HGF nuclei were visualized with 4′,6-diamidino-2-phenylindole (DAPI, Sigma-Aldrich, USA) staining. HGFs were observed with confocal laser scanning microscopy (LSM-880, Carl Zeiss, Oberkochen, Germany).

### 2.6. Western Blotting Analysis

HGFs were pretreated with TNF-*α* (10 ng/ml) and IL-1*β* (2 ng/ml) for 24 h, with increasing concentrations of CORM-3. Unstimulated cells were used as a control. HGFs were washed three times with ice-cold PBS and lysed with RIPA lysis buffer (Beyotime Institute of Biotechnology, Jiangsu, China). After 30 min on ice, the cells were separated by centrifugation at a speed of 12,000 × g at 4°C for 5 min. The protein samples were quantified using a BCA kit (Solarbio, Beijing, China), and 30 *μ*g of each sample was mixed with a quarter volume of 5x SDS-PAGE loading buffer (Solarbio) and boiled for 5 min to denature. Then, whole-cell protein samples were isolated by 10% sodium dodecyl sulfate- (SDS-) polyacrylamide gel electrophoresis (Gel Preparation Kit, Boster Biological Technology, Pleasanton, CA, USA) and transferred to polyvinylidene fluoride membranes (0.45 *μ*M) by electrophoresis. The membranes were blocked with 5% nonfat dry milk powder in PBS for 1 h at room temperature. Then, the membranes were incubated overnight at 4°C with the following specific primary antibodies: anti-human ZO-1 rabbit monoclonal antibody (1 : 1000 dilution), anti-human VE-cadherin rabbit polyclonal antibody (1 : 1000 dilution), anti-human heme oxygenase-1 (HO-1) rabbit monoclonal antibody (1 : 10000 dilution), anti-human beta-catenin rabbit monoclonal antibody (1 : 5000 dilution), anti-human GAPDH rabbit polyclonal antibody (1 : 5000 dilution), and anti-human *β*-actin rabbit polyclonal antibody (1 : 5000 dilution). After three extensive washes, the membranes were incubated with the appropriate horseradish peroxidase-conjugated goat anti-rabbit IgG (1 : 5000 dilution) or horseradish peroxidase-conjugated goat anti-mouse IgG (1 : 5000 dilution) for 1 h at room temperature. After three extensive washes with Tris-buffered saline Tween-20 (TBST), the immunoreactive bands were detected with enhanced chemiluminescence (Millipore, Bedford, IL, USA) according to the manufacturer's instructions and quantified using Imaging J software (NIH, USA).

### 2.7. RNA Isolation and Reverse-Transcriptase-Polymerase Chain Reaction (RT-PCR)

HGFs were cultured in 6-well plates (200,000 cells/well) containing various stimulants for 24 h with increasing concentrations of CORM-3. Unstimulated cells were used as a control. For the first part of the experiment, HGFs were divided into six groups: the control group, TNF-*α* (10 ng/ml)+IL-1*β* (2 ng/ml) group, CORM-3 (100 *μ*M)+TNF-*α* (10 ng/ml)+IL-1*β* (2 ng/ml) group, CORM-3 (200 *μ*M)+TNF-*α* (10 ng/ml)+IL-1*β* (2 ng/ml) group, and CORM-3 (400 *μ*M)+TNF-*α* (10 ng/ml)+IL-1*β* (2 ng/ml) group.

For the second part of the experiment, HGFs were divided into seven groups as follows: the control group, TNF-*α* (10 ng/ml)+IL-1*β* (2 ng/ml) group, CORM-3 (200 *μ*M)+TNF-*α* (10 ng/ml)+IL-1*β* (2 ng/ml) group, HO-1 siRNA+CORM-3 (200 *μ*M)+TNF-*α* (10 ng/ml)+IL-1*β* (2 ng/ml) group, HO-1 siRNA+TNF-*α* (10 ng/ml)+IL-1*β* (2 ng/ml) group, scrambled siRNA+CORM-3 (200 *μ*M)+TNF-*α* (10 ng/ml)+IL-1*β* (2 ng/ml) group, and scrambled siRNA+TNF-*α* (10 ng/ml)+IL-1*β* (2 ng/ml) group. All the groups were incubated for 24 h.

Total RNA was isolated from cultured cells using Trizol Reagent (Invitrogen, Carlsbad, USA) according to the manufacturer's instructions. One microgram of total RNA was reverse transcribed into complementary DNA with the PrimeScript RT reagent Kit with gDNA Eraser (TaKara, Kusatsu, Japan) according to the manufacturer's protocol. Quantitative RT-PCR was performed with TB Green Premix Ex Taq II (TaKara, Kusatsu, Japan) on an Applied Biosystems StepOnePlus Real-Time PCR System. The primers used were as follows: ZO-1 forward, 5′-GAAATACCTGACGGTGCTGC-3′ and reverse, 5′-GAGGATGGCGTTACCCACAG-3′; VE-cadherin forward, 5′-AAGGACACTGGCGAAAACCT-3′ and reverse, 5′-ACGCATTGAACAACCGATGC-3′; *β*-catenin forward, 5′-GGCTTGGAATGAGACTGCTG-3′ and reverse, 5′-GGTCCATACCCAAGGCATCC-3′; HO-1 forward, 5′-AGGCCAAGACTGCGTTCCT-3′, and reverse, 5′-AACTGTCGCCACCAGAAAGCTGAG-3′; and GAPDH forward, 5′-GCACCGTCAAGGCTGAGAAC-3′ and reverse, 5′-TGGTGAAGACGCCAGTGGA-3′. The real-time quantitative PCR cycling conditions used were as follows: initial denaturation at 95°C for 30 s, then 40 cycles of denaturation at 95°C for 5 s, annealing at 60°C for 34 s, and extension for 45 s at 72°C. The cycle threshold values for target gene expression were normalized to those of glyceraldehyde-3-phosphate dehydrogenase (GAPDH), and expression was calculated using the 2^-*ΔΔ*Ct^ method.

### 2.8. Transient Transfection

The HO-1 siRNA target sequences for HGFs were as follows: forward, 5′-GGGUCCUUACAUUCAGCUUTT-3′, and reverse, 5′-AAGCUGAGUGUAAGGACCCTT-3′. Seven groups were established for the second part of the experiment. HGFs were seeded and cultured in control medium in 6-well plates at a density of 150,000 cells/well. HGFs were transfected with 5 *μ*l siRNA and Lipofectamine 2000 Transfection Reagent (Invitrogen, Thermo Fisher Scientific, Inc.) for 6 h according to the manufacturer's protocol. The cells were cultured in Opti-MEM Reduced Serum Medium (Gibco, Thermo Fisher Scientific, Inc.) and transiently transfected with scrambled siRNA or HO-1 siRNA, followed by TNF-*α* (10 ng/ml) and IL-1*β* (2 ng/ml) treatments in the presence or absence of CORM-3.

### 2.9. Statistical Analysis

The data were obtained from at least three independent experiments performed in triplicate. The significance of different groups was assessed by GraphPad Prism 6 software (GraphPad, CA, USA). Statistical significance was determined using one-way ANOVA followed by Tukey's multiple comparison test and *p* values of less than 0.05 were considered significant.

## 3. Results

### 3.1. Effects of CORM-3 on HGF Proliferation

**Figure 1:**
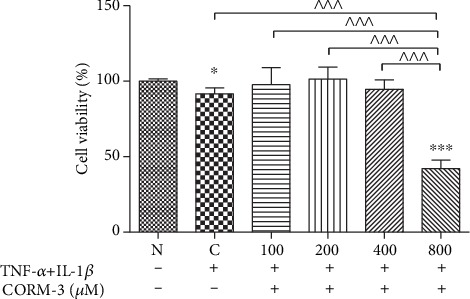
Effect of CORM-3 on the viability of human gingival fibroblasts (HGFs). HGFs were stimulated with 0, 100, 200, 400, and 800 *μ*M CORM-3 and 10 ng/ml TNF-*α* and 2 ng/m IL-1*β* for 24 h. Then, cell viability was assessed by Cell Counting Kit-8 (CCK-8) assays. The values were obtained from at least three independent experiments performed in triplicate, where ^∗^*p* < 0.05 and ^∗∗∗^*p* < 0.001 versus normal and noninduced HGFs and ^^^^^*p* < 0.001 compared between two groups, according to ANOVA followed by Tukey's multiple comparison test. C: TNF-*α*- and IL-1*β*-induced cells; N: normal and noninduced HGFs. CORM-3: carbon monoxide-releasing molecule-3; OD: optical density; TNF-*α*: tumor necrosis factor-*α*; IL-1*β*: interleukin-1*β*.

Figure 1 shows the effect of CORM-3 on HGF proliferation. HGFs were incubated with different concentrations of CORM-3 for 24 h. Cell proliferation was assessed by a CCK-8 assay. Concurrent stimulation of 10 ng/ml TNF-*α* and 2 ng/ml IL-1*β* decreased the cell viability significantly in the absence of CORM-3 (*p* < 0.05). While in the presence of CORM-3 at the concentration of 100, 200, and 400 *μ*M, respectively, the cell viability maintained the same level as that of the nonstimulated cells. CORM-3 at 800 *μ*M significantly inhibited HGF proliferation compared with normal and noninduced HGF proliferation (*p* < 0.001). Therefore, CORM-3 was used at 400 *μ*M or less for the subsequent assays.

### 3.2. TNF-*α*- and IL-1*β*-Induced High HGF Monolayer Permeability Was Blocked by CORM-3

**Figure 2:**
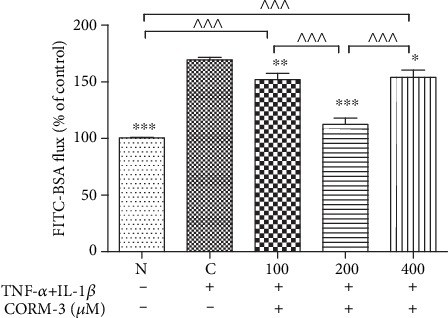
Effect of CORM-3 on HGF monolayer permeability in response to TNF-*α* and IL-1*β*. HGFs were stimulated with 10 ng/ml TNF-*α* and 2 ng/ml IL-1*β* with increasing concentrations of CORM-3 for 24 h, followed by assessment for FITC-BSA flux. The values were obtained from at least three independent experiments performed in triplicate, where ^∗^*p* < 0.05, ^∗∗^*p* < 0.01, and ^∗∗∗^*p* < 0.001 versus TNF-*α*- and IL-1*β*-induced cells and ^^^^^*p* < 0.001 compared between two groups, according to ANOVA followed by Tukey's multiple comparison test. C: TNF-*α*- and IL-1*β*-induced cells; N: normal and noninduced HGFs; CORM-3: carbon monoxide-releasing molecule-3; TNF-*α*: tumor necrosis factor-*α*; IL-1*β*: interleukin-1*β*; FITC-BSA: fluorescein isothiocyanate-bovine serum albumin.

Gingival edema is a hallmark of periodontitis that can be induced by acute inflammation. Apart from cellular changes, most edema results from impaired barrier integrity indicated by increased epithelial monolayer permeability. Changes in the permeability of HGF monolayers to CORM-3 were assessed by measuring FITC-BSA flux following 24 h of incubation with or without TNF-*α* and IL-1*β*, CORM-3.  Figure 2 shows that the inflammatory mediators TNF-*α* and IL-1*β* significantly increased the permeability of the HGF monolayer (*p* ≤ 0.001), and CORM-3 was able to reduce this effect by 82.8 ± 6.8% (*p* ≤ 0.001) at 200 *μ*M, by 25.4 ± 5.7% (*p* ≤ 0.01) at 100 *μ*M, and by 22.3 ± 7% (*p* ≤ 0.05) at 400 *μ*M. The greatest inhibitory effect of CORM-3 on permeability was observed at 200 *μ*M (*p* ≤ 0.001).

### 3.3. Immunofluorescence Staining

**Figure 3:**
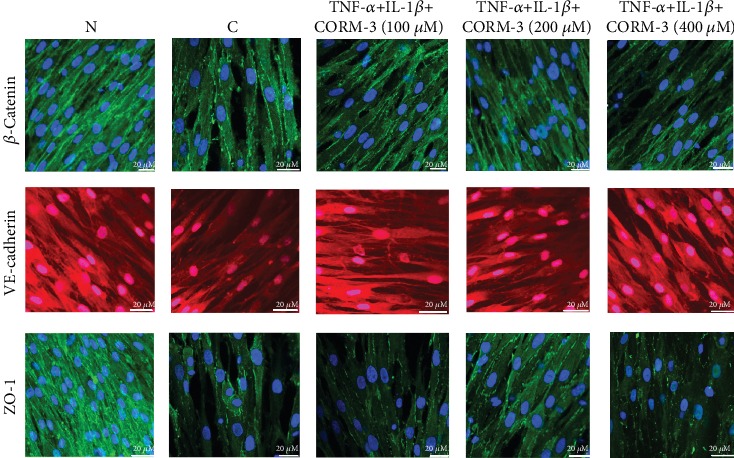
Location and expression changes of *β*-catenin, VE-cadherin, and ZO-1 in HGFs treated with CORM-3 in response to TNF-*α* and IL-1*β* stimulation. HGFs were stimulated with 10 ng/ml TNF-*α* and 2 ng/ml IL-1*β* with increasing concentrations of CORM-3 for 24 h, followed by confocal immunofluorescence assessment (original magnification ×400). DAPI (blue) was used for nuclear staining. TNF-*α* and IL-1*β* induction resulted in a decrease in *β*-catenin, VE-cadherin, and ZO-1 expressions in HGFs. The pictures are representative of three independent experiments. Scale bar = 20 *μ*M. C: TNF-*α*- and IL-1*β*-induced cells; N: normal and noninduced HGFs; ZO-1: zona occludens-1; CORM-3: carbon monoxide-releasing molecule-3; TNF-*α*: tumor necrosis factor-*α*; IL-1*β*: interleukin-1*β*; DAPI, 4′,6-diamidino-2-phenylindole.

VE-cadherin and *β*-catenin are major transmembrane adherens junction molecules in vascular endothelial cells. Zona occludens-1 (ZO-1) is a major transmembrane tight junction molecule in vascular endothelial cells. These factors represent crucial determinants of cell barrier integrity. Immunostaining for VE-cadherin, ZO-1, and *β*-catenin showed a linear staining pattern at the cell borders under control conditions in confluent HGF cell monolayers (Figure 3). Incubation with TNF-*α* and IL-1*β* for 24 h led to intercellular gap formation and pronounced loss of VE-cadherin, ZO-1, and *β*-catenin staining at the cell borders. Incubation with increasing CORM-3 concentrations prevented the loss of VE-cadherin, ZO-1, and *β*-catenin to different degrees. The inhibitory effects of CORM-3 on VE-cadherin, ZO-1, and *β*-catenin loss also showed dose dependency. The most obvious protective effects were observed at a concentration of 200 *μ*M for every junction molecule (Figure 3).

### 3.4. CORM-3 Prevented Junctional Complex Molecule Disruption

**Figure 4:**
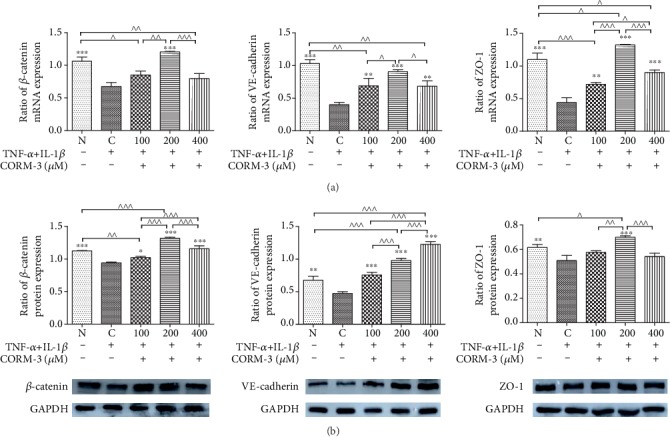
Effects of CORM-3 on the expression of junctional complex molecules in HGFs following TNF-*α* and IL-1*β* induction. (a) Effects of CORM-3 on *β*-catenin, VE-cadherin, and ZO-1 mRNA expression in HGFs. (b) Protein expression of junctional complex molecules in HGFs following TNF-*α* and IL-1*β* induction. HGFs were stimulated with TNF-*α* (10 ng/ml) and IL-1*β* (2 ng/ml) and treated with increasing CORM-3 concentrations for 24 h. Total RNA or protein was extracted and analyzed using RT-qPCR or western blot analysis. The values were obtained from at least three independent experiments performed in triplicate, where ^∗^*p* < 0.05, ^∗∗^*p* < 0.01, and ^∗∗∗^*p* < 0.001 versus TNF-*α*- and IL-1*β*-induced cells and ^^^*p* < 0.05, ^^^^*p* < 0.01, and ^^^^^*p* < 0.001 compared between two groups, according to the ANOVA followed by Tukey's multiple comparison test. C: TNF-*α*- and IL-1*β*-induced cells; N: normal and noninduced HGF; HGFs: human gingival fibroblasts; CORM-3: carbon monoxide-releasing molecule-3; TNF-*α*: tumor necrosis factor-*α*; IL-1*β*: interleukin-1*β*.

Junctional complex molecules play major roles in cell membrane barrier integrity. Junctional complexes consist of tight junctions, which regulate paracellular transport, and adherens junctions, which are mainly responsible for cell-to-cell adhesion. Because CORM-3 was able to suppress TNF-*α* (10 ng/ml)- and IL-1*β* (2 ng/ml)-induced high permeability, whether these effects are related to changes in the expression of the junctional complex molecules VE-cadherin, ZO-1, and *β*-catenin was elucidated. Incubation with TNF-*α* and IL-1*β* induced an increase in cell permeability (Figure 2). After TNF-*α* and IL-1*β* were added to HGFs for 24 h, VE-cadherin, ZO-1, and *β*-catenin were significantly decreased at the gene and protein levels compared with those in normal and noninduced cells. After CORM-3 was added, the VE-cadherin, ZO-1, and *β*-catenin levels were reversed significantly at 200 *μ*M (Figure 4).

### 3.5. CORM-3 Promotes HO-1 Expression in HGFs Incubated with or without TNF-*α* and IL-1*β*

**Figure 5:**
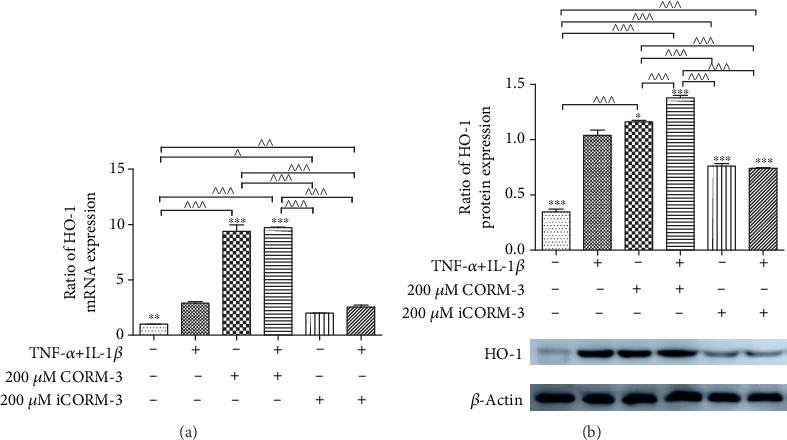
Effects of CORM-3 on HO-1 mRNA and protein expression in HGFs incubated with TNF-*α* and IL-1*β*. (a) HGFs were divided into six groups: control; TNF-*α*+IL-1*β*, in which HGFs were incubated with 10 ng/ml TNF-*α* and 2 ng/ml IL-1*β* for 24 h; CORM-3, in which HGFs were treated with 200 *μ*M CORM-3 for 24 h; CORM-3+TNF-*α*+IL-1*β*, in which HGFs were treated with 200 *μ*M CORM-3 and 10 ng/ml TNF-*α* and 2 ng/ml IL-1*β* for 24 h; iCORM-3, in which HGFs were treated with 200 *μ*M inactivated CORM-3 for 24 h; and iCORM-3+TNF-*α*+IL-1*β*, in which cells were incubated with 200 *μ*M inactivated CORM-3 and 10 ng/ml TNF-*α* and 2 ng/ml IL-1*β* for 24 h. HO-1 mRNA expression levels were determined by RT-qPCR. (b) Protein expression levels of HO-1 were determined by western blotting. *β*-Actin was used to demonstrate equal sample loading. The values were obtained from at least three independent experiments performed in triplicate, where ^∗^*p* < 0.05, ^∗∗^*p* < 0.01, and ^∗∗∗^*p* < 0.001 versus TNF-*α*- and IL-1*β*-induced cells and ^^^*p* < 0.05, ^^^^*p* < 0.01, and ^^^^^*p* < 0.001 compared between two groups, according to ANOVA followed by Tukey's multiple comparison test. HO-1: heme oxygenase-1; CORM-3: carbon monoxide-releasing molecule-3; TNF-*α*: tumor necrosis factor-*α*; IL-1*β*: interleukin-1*β*; HGFs: human gingival fibroblasts.

HGFs were incubated with CORM-3 (200 *μ*M) and with or without TNF-*α* (10 ng/ml) and IL-1*β* (2 ng/ml) for 24 h. Then, HO-1 mRNA and protein expression levels were examined by RT-PCR and western blotting. The mRNA and protein expression levels of HO-1 induced by CORM-3 were significantly increased compared with those in the control group (Figure 5). Deactivated CORM-3 did not induce HO-1 expression, which indicated that the effect of CORM-3 mainly resulted in CO. The results showed that TNF-*α*- and IL-1*β*-induced HO-1 expression levels were comparable to those in the control group. Compared with control conditions, CORM-3 significantly increased the mRNA and protein expressions of HO-1 in HGFs (Figure 5). On the other hand, deactivated CORM-3 did not have an inductive effect on HO-1. CORM-3 could promote HO-1 expression by releasing CO.

### 3.6. Effect of HO-1 Silencing on the Expression of Junctional Complex Molecules after Incubation with TNF-*α* and IL-1*β* and with or without CORM-3 Pretreatment

**Figure 6:**
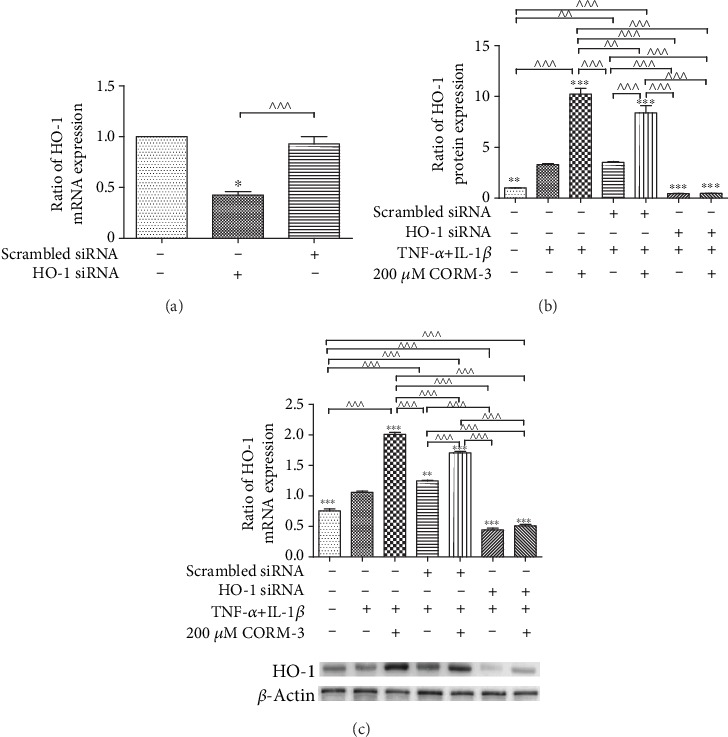
HO-1 expression in HGFs transfected with HO-1 siRNA. (a) HGFs were transiently transfected with HO-1 siRNA. Scrambled siRNA was used as control. mRNA expression of HO-1 was detected by RT-qPCR. (b) HGFs were divided into seven groups: control, TNF-*α*+IL-1*β*, in which HGFs were incubated with 10 ng/ml TNF-*α* and 2 ng/ml IL-1*β* for 24 h; CORM-3+TNF-*α*+IL-1*β*, in which HGFs were treated with 200 *μ*M CORM-3, 10 ng/ml TNF-*α* and 2 ng/ml IL-1*β* for 24 h; scrambled siRNA+TNF-*α*+IL-1*β*, in which cells were transiently transfected with scrambled siRNA 6 h prior to incubation with 10 ng/ml TNF-*α* and 2 ng/ml IL-1*β* for 24 h; scrambled siRNA+TNF-*α*+IL-1*β*+CORM-3, in which cells were transiently transfected with scrambled siRNA for 6 h were incubated with 10 ng/ml TNF-*α*, 2 ng/ml IL-1*β*, and 200 *μ*M CORM-3 for 24 h; HO-1 siRNA+TNF-*α*+IL-1*β*, in which cells were transiently transfected with HO-1 siRNA 6 h prior to incubation with 10 ng/ml TNF-*α* and 2 ng/ml IL-1*β* for 24 h; and HO-1 siRNA+TNF-*α*+IL-1*β*+CORM-3, in which cells were transiently transfected with HO-1 siRNA 6 h prior to incubation with 10 ng/ml TNF-*α*, 2 ng/ml IL-1*β*, and 200 *μ*M CORM-3 for 24 h. HO-1 mRNA expression levels were determined by RT-qPCR. (c) Protein expression levels of HO-1 were determined by western blotting. *β*-Actin was used to demonstrate equal sample loading. The values were obtained from at least three independent experiments performed in triplicate, where ^∗^*p* < 0.05, ^∗∗^*p* < 0.01, and ^∗∗∗^*p* < 0.001 versus TNF-*α*- and IL-1*β*-induced cells and ^^^^*p* < 0.01 and ^^^^^*p* < 0.001, compared between two groups, according to ANOVA followed by Tukey's multiple comparison test. HO-1: heme oxygenase-1; CORM-3: carbon monoxide-releasing molecule-3; siRNA: small interfering RNA; TNF-*α*: tumor necrosis factor-*α*; IL-1*β*: interleukin-1*β*; HGFs: human gingival fibroblasts.

**Figure 7:**
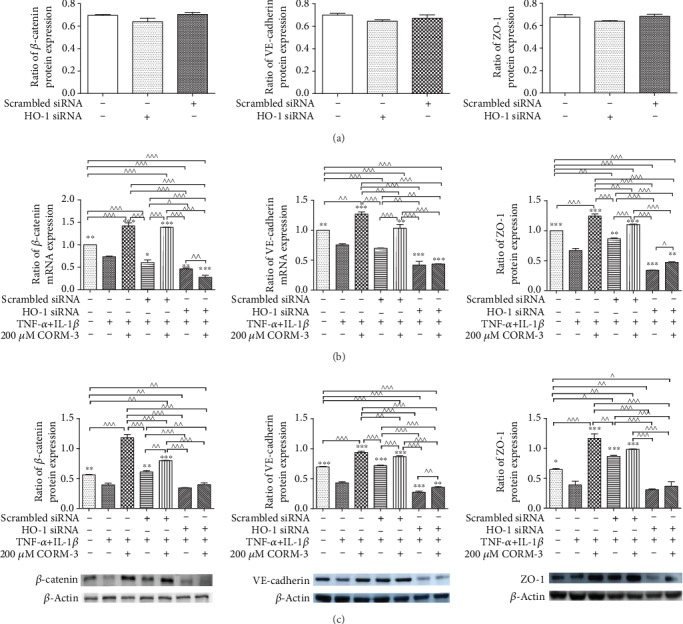
Expression changes of *β*-catenin, VE-cadherin, and ZO-1 in HGFs transfected with HO-1 siRNA. (a) HGFs were transiently transfected with HO-1 siRNA or scrambled siRNA. HGFs without transfection were used as control. mRNA expression of tight and adherens junctions was detected by RT-qPCR. (b) HGFs were divided into seven groups: control; TNF-*α*+IL-1*β*, in which HGFs were incubated with 10 ng/ml TNF-*α* and 2 ng/ml IL-1*β* for 24 h; CORM-3+TNF-*α*+IL-1*β*, in which HGFs were treated with 200 *μ*M CORM-3, 10 ng/ml TNF-*α*, and 2 ng/ml IL-1*β* for 24 h; scrambled siRNA+TNF-*α*+IL-1*β*, in which cells were transiently transfected with scrambled siRNA 6 h prior to incubation with 10 ng/ml TNF-*α* and 2 ng/ml IL-1*β* for 24 h; scrambled siRNA+TNF-*α*+IL-1*β*+CORM-3, in which cells transiently transfected with scrambled siRNA for 6 h were incubated with 10 ng/ml TNF-*α*, 2 ng/ml IL-1*β*, and 200 *μ*M CORM-3 for 24 h; HO-1 siRNA+TNF-*α*+IL-1*β*, in which cells were transiently transfected with HO-1 siRNA 6 h prior to incubation with 10 ng/ml TNF-*α* and 2 ng/ml IL-1*β* for 24 h; HO-1 siRNA+TNF-*α*+IL-1*β*+CORM-3, in which cells were transiently transfected with HO-1 siRNA 6 h prior to incubation with 10 ng/ml TNF-*α*, 2 ng/ml IL-1*β*, and 200 *μ*M CORM-3 for 24 h. HO-1 mRNA expression levels were determined by RT-qPCR. (c) Protein expression levels of HO-1 were determined by western blotting. *β*-Actin was used to demonstrate equal sample loading. The values were obtained from at least three independent experiments performed in triplicate, where ^∗^*p* < 0.05, ^∗∗^*p* < 0.01, and ^∗∗∗^*p* < 0.001 versus TNF-*α*- and IL-1*β*-induced cells and ^^^*p* < 0.05, ^^^^*p* < 0.01, and ^^^^^*p* < 0.001 compared between two groups, according to ANOVA followed by Tukey's multiple comparison test. HO-1: heme oxygenase-1; CORM-3: carbon monoxide-releasing molecule-3; siRNA: small interfering RNA; TNF-*α*: tumor necrosis factor-*α*; IL-1*β*: interleukin-1*β*; HGFs: human gingival fibroblasts.

To examine whether the regulatory effect of CORM-3 on HGFs was mediated by HO-1, HO-1 siRNA was transfected into HGFs. As shown in Figure 6(a), HO-1 siRNA transfection decreased the mRNA expression of HO-1 significantly, while scrambled siRNA transfection had no effect on HO-1 expression. In other experiments, HGFs were transfected with HO-1 small interfering RNA (siRNA) for 6 h and then incubated with TNF-*α* and IL-1*β* and with or without CORM-3 for 24 h. The mRNA and protein expression level changes in HO-1 were significantly abolished by HO-1 siRNA transfection (Figure 6). HO-1 siRNA or scrambled siRNA transfected cells were used to clarify the influence of HO-1 or scrambled siRNA on the expression of VE-cadherin, ZO-1, and *β*-catenin. The results showed that HO-1 siRNA transfection suppressed the anti-inflammatory effects of CORM-3 (Figure 7), and VE-cadherin, ZO-1, and *β*-catenin expression levels were not increased by CORM-3. The results are shown by the mRNA (Figure 7(a)) and protein (Figure 7(b)) levels. The expression levels of VE-cadherin, ZO-1, and *β*-catenin were lower in the HO-1 siRNA+CORM-3+TNF-*α*+IL-1*β* group than in the control group (*p* < 0.05) but were not significantly different than those in the HO-1 siRNA+TNF-*α*+IL-1*β* group. The results that CORM-3 was no longer effective in inducing the anti-inflammatory effects of junctional complex molecule VE-cadherin, ZO-1, and *β*-catenin gene and protein expression after HO-1 siRNA transfection suggested that the regulatory effect of CORM-3 was mediated by mainly the HO-1 pathway.

## 4. Discussion

Recent studies have demonstrated the anti-inflammatory effects of CORM-3 on a number of cells, such as vascular endothelial cells, macrophages, and monocytes [22–24]. However, the effect of CORM-3 on decreasing proinflammatory cytokine-stimulated HGF barrier permeability by downregulating tight junction molecules (ZO-1) and adherens junction molecules (*β*-catenin and VE-cadherin) has not been delineated. In the present study, the effect of CORM-3 on TNF-*α*- and IL-1*β*-induced inflammatory responses and the role of HO-1 in the process were investigated.

HGFs were selected for use in the present study because they are the group of cells that are most highly affected by TNF-*α* and IL-1*β* in periodontitis. They also play a vital role in many processes, so changes in the barrier integrity and function due to inflammatory mediators will lead to impaired periodontal function [25, 26]. It is well known that periodontitis is a complicated pathological inflammatory process, which means that the expression or secretion of various proinflammatory cytokines, such as chemokines and adherens molecules, may increase simultaneously in the local environment. It is well known that TNF-*α* and IL-1*β* are two of the most important inflammatory cytokines involved in periodontitis, and both of these molecules have been shown to enhance leukocyte adhesion by increasing barrier permeability and downregulating tight and adherens junction molecule expressions on epithelial cells [7, 27, 28]. The effects of TNF-*α* and IL-1*β* on HGF barrier integrity and function are also supported by *in vitro* studies^21^. Herein, we report that CORM-3 can suppress TNF-*α*- and IL-1*β*-induced changes in HGF permeability and tight and adherens junction expressions. These findings suggest that CORM-3 exerts beneficial effects on HGF barrier integrity and function in response to inflammation.

The results of the study suggested that when HGFs were exposed to TNF-*α* and IL-1*β*, VE-cadherin, ZO-1, and *β*-catenin production decreased. Furthermore, it was found that incubation with CORM-3 effectively downregulated TNF-*α*- and IL-1*β*-induced VE-cadherin, ZO-1, and *β*-catenin levels in HGFs. In contrast, inactivated CORM-3 (iCORM-3), which cannot release CO, did not attenuate TNF-*α*- and IL-1*β*-induced inflammatory cytokine expressions in HGFs. Thus, it may be hypothesized that CORM-3 exerts anti-inflammatory effects via CO release. CO is a metabolite of HO-catalyzed heme degradation, and this is the primary cellular source of endogenous CO [19], which excludes the role of toxic molecules as widespread signaling molecules in regulating cell function and communication. As a water-soluble CORM, CORM-3 is an effective carrier to deliver CO and modulate many endogenous reactions [24, 29]. In addition, many animal studies have found that CORMs have protective effects in the heart and lungs [30, 31], including hyperacute endotoxic shock, pulmonary inflammation, postoperative ileus, airway hyperresponsiveness, and pulmonary hypertension [32–35].

The heme oxygenase (HO) system is a microsomal enzyme system that exists widely in humans and mammals. To date, three isoforms of HO have been reported: HO-1, HO-2, and HO-3. HO-1 is reported to be an inducible enzyme [36, 37]. In addition, HO-1 plays vital roles in regulating cell growth and differentiation and in controlling proinflammatory cytokines and other stresses [38–40]. The present study demonstrated that TNF-*α* and IL-1*β* induced HO-1 expression in HGFs, and CORM-3 induced higher HO-1 expression than the combination of TNF-*α* and IL-1*β*. The present study hypothesized that HO-1 induction by CORM-3 in HGFs may be responsible for the anti-inflammatory effects of CORM-3 against the inflammation induced by TNF-*α* and IL-1*β*. The present study clearly showed that CORM-3 significantly increased HO-1 expression during the anti-inflammatory process. However, CORM-3 lost its anti-inflammatory effect after HO-1 siRNA was transfected into HGFs, which indicated that the effect of CORM-3 relied on HO-1 [41].

Periodontal disease is a major global oral health problem, but there are currently no therapies available to limit its progression [42–44]. The results of the present study demonstrate for the first time that the anti-inflammatory effects of CORM-3 occur via HO-1-dependent pathways in periodontal disease models, and these pathways are mediated through VE-cadherin, ZO-1, and *β*-catenin expressions. Overall, the results suggest that CORM-3 may have promising potential therapeutic value for the treatment of inflammatory periodontal disease. The exact mechanisms of the protective effects of CORM-3 remain to be determined. In addition, the beneficial effects of CORM-3 in *in vivo* settings require further investigation in the future.

CORMs, the chemical CO-donor compounds, as an alternative approach to the administration of CO gas, have been shown to have many beneficial effects, even in a primate animal model [45]. In particular, the application of CORMs is likely to more effectively bypass the biologic trap represented by deoxyhemoglobin, which gives rise to less COHb buildup typical of inhalation CO. Recently, hybrid molecule-termed HYCOs by conjugating a CORM with various nuclear factor erythroid 2-related factor 2 (Nrf2) activators has been designed [46]. These finds suggest a more promising future in clinical application of CORMs.

## Data Availability

All data used to support the findings of this study are included within the article.

## References

[1] Wang X., Shi L., Ying X. (2019). Preliminary study on the effects of smoking on gingival microcirculation in chronic periodontitis. *Hua Xi Kou Qiang Yi Xue Za Zhi*.

[2] Graves D. T., Cochran D. (2003). The contribution of interleukin-1 and tumor necrosis factor to periodontal tissue Destruction. *Journal of Periodontology*.

[3] Dzink J. L., Tanner A. C., Haffajee A. D., Socransky S. S. (1985). Gram negative species associated with active destructive periodontal lesions. *Journal of Clinical Periodontology*.

[4] Clark R., Zwicker S., Bureik D., Johannsen G., Boström E. A. (2020). Expression of colony-stimulating factor 1 and interleukin-34 in gingival tissue and gingival fibroblasts from periodontitis patients and controls. *Journal of Periodontology*.

[5] Hegde R., Awan K. H. (2019). Effects of periodontal disease on systemic health. *Disease-a-Month*.

[6] Kang S. K., Park Y. D., Kang S. I. (2015). Role of resistin in the inflammatory response induced by nicotine plus lipopolysaccharide in human periodontal ligament cells in vitro. *Journal of Periodontal Research*.

[7] Sim T., Harith H., Tham C. (2018). The protective effects of a synthetic geranyl acetophenone in a cellular model of TNF-*α*-Induced pulmonary epithelial barrier dysfunction. *Molecules*.

[8] Boström E. A., Kindstedt E., Sulniute R. (2015). Increased eotaxin and MCP-1 levels in serum from individuals with periodontitis and in human gingival fibroblasts exposed to pro-inflammatory cytokines. *PLoS One*.

[9] Brune K., Frank J., Schwingshackl A., Finigan J., Sidhaye V. K. (2015). Pulmonary epithelial barrier function: some new players and mechanisms. *American Journal of Physiology-Lung Cellular and Molecular Physiology*.

[10] Vogelmann R., Amieva M. R., Falkow S., Nelson W. J. (2004). Breaking into the epithelial apical-junctional complex--news from pathogen hackers. *Current Opinion in Cell Biology*.

[11] Gueron G., Giudice J., Valacco P. (2014). Heme-oxygenase-1 implications in cell morphology and the adhesive behavior of prostate cancer cells. *Oncotarget*.

[12] Bussolati B., Ahmed A., Pemberton H. (2004). Bifunctional role for VEGF-induced heme oxygenase-1 in vivo: induction of angiogenesis and inhibition of leukocytic infiltration. *Blood*.

[13] Gueron G., De Siervi A., Ferrando M. (2009). Critical role of endogenous heme oxygenase 1 as a tuner of the invasive potential of prostate cancer cells. *Molecular Cancer Research*.

[14] Pi S. H., Jeong G. S., Oh H. W. (2010). Heme oxygenase-1 mediates nicotine- and lipopolysaccharide-induced expression of cyclooxygenase-2 and inducible nitric oxide synthase in human periodontal ligament cells. *Journal of Periodontal Research*.

[15] Zhang X., Shan P., Otterbein L. E. (2003). Carbon monoxide inhibition of apoptosis during ischemia-reperfusion lung injury is dependent on the p38 mitogen-activated protein kinase pathway and involves caspase 3. *Journal of Biological Chemistry*.

[16] Otterbein L. E., Bach F. H., Alam J. (2000). Carbon monoxide has anti-inflammatory effects involving the mitogen-activated protein kinase pathway. *Nature Medicine*.

[17] Matsumoto M., Makino Y., Tanaka T. (2003). Induction of renoprotective gene expression by cobalt ameliorates ischemic injury of the kidney in rats. *Journal of the American Society of Nephrology*.

[18] Sikorski E. M., Hock T., Hill-Kapturczak N., Agarwal A. (2004). The story so far: molecular regulation of the heme oxygenase-1 gene in renal injury. *American Journal of Physiology. Renal Physiology*.

[19] Lin C. C., Yang C. C., Hsiao L. D., Chen S. Y., Yang C. M. (2017). Heme oxygenase-1 induction by carbon monoxide releasing molecule-3 suppresses Interleukin-1*β*-Mediated Neuroinflammation. *Frontiers in Molecular Neuroscience*.

[20] Masini E., Vannacci A., Failli P. (2008). A carbon monoxide-releasing molecule (CORM-3) abrogates polymorphonuclear granulocyte-induced activation of endothelial cells and mast cells. *The FASEB Journal*.

[21] Song H., Zhao H., Qu Y. (2011). Carbon monoxide releasing molecule-3 inhibits concurrent tumor necrosis factor-*α*- and interleukin-1*β*-induced expression of adhesion molecules on human gingival fibroblasts. *Journal of Periodontal Research*.

[22] Zhang L. M., Zhang D. X., Fu L. (2019). Carbon monoxide-releasing molecule-3 protects against cortical pyroptosis induced by hemorrhagic shock and resuscitation via mitochondrial regulation. *Free Radical Biology & Medicine*.

[23] Portal L., Morin D., Motterlini R., Ghaleh B., Pons S. (2019). The CO-releasing molecule CORM-3 protects adult cardiomyocytes against hypoxia-reoxygenation by modulating pH restoration. *European Journal of Pharmacology*.

[24] Bihari A., Chung K. (. A.)., Cepinskas G., Sanders D., Schemitsch E., Lawendy A.‐. R. (2019). Carbon monoxide-releasing molecule-3 (CORM-3) offers protection in an in vitro model of compartment syndrome. *Microcirculation*.

[25] Urnowey S., Ansai T., Bitko V., Nakayama K., Takehara T., Barik S. (2019). Retraction note: temporal activation of anti- and pro-apoptotic factors in human gingival fibroblasts infected with the periodontal pathogen, Porphyromonas gingivalis: potential role of bacterial proteases in host signalling. *BMC Microbiology*.

[26] Lu J., Ren B., Wang L., Li M., Liu Y. (2019). Preparation and evaluation of IL-1ra-loaded dextran/PLGA microspheres for inhibiting periodontal inflammation in vitro. *Inflammation*.

[27] Brazee P. L., Soni P. N., Tokhtaeva E. (2017). FXYD5 is an essential mediator of the inflammatory response during lung Injury. *Frontiers in Immunology*.

[28] Wang W., Guan W. J., Huang R. Q. (2016). Carbocisteine attenuates TNF-*α*-induced inflammation in human alveolar epithelial cells in vitro through suppressing NF-*κ*B and ERK1/2 MAPK signaling pathways. *Acta Pharmacologica Sinica*.

[29] Carvalho S. M., Marques J., Romão C. C., Saraiva L. M. (2019). Metabolomics ofEscherichia coliTreated with the antimicrobial carbon monoxide-releasing molecule CORM-3 reveals tricarboxylic acid cycle as major target. *Antimicrob Agents Chemother*.

[30] Pak O., Bakr A. G., Gierhardt M. (2016). Effects of carbon monoxide-releasing molecules on pulmonary vasoreactivity in isolated perfused lungs. *Journal of Applied Physiology*.

[31] Zhuk S., Smith O., Thondapu V., Halupka K., Moore S. (2020). Using contrast motion to generate patient specific blood flow simulations during invasive coronary angiography. *Journal of Biomechanical Engineering*.

[32] Li J., Song L., Hou M., Wang P., Wei L., Song H. (2018). Carbon monoxide releasing molecule‑3 promotes the osteogenic differentiation of rat bone marrow mesenchymal stem cells by releasing carbon monoxide. *International Journal of Molecular Medicine*.

[33] Kumada Y., Takahashi T., Shimizu H. (2019). Therapeutic effect of carbon monoxide-releasing molecule-3 on acute lung injury after hemorrhagic shock and resuscitation. *Experimental and Therapeutic Medicine*.

[34] de Rivero Vaccari J. P. (2019). Carbon monoxide releasing molecule-3 inhibits inflammasome activation: a potential therapy for spinal cord injury. *eBioMedicine*.

[35] Zheng G., Zhan Y., Wang H. (2019). Carbon monoxide releasing molecule-3 alleviates neuron death after spinal cord injury via inflammasome regulation. *eBioMedicine*.

[36] Pan Y., Song J., Ma L. (2018). Carbon monoxide releasing molecule 3 inhibits osteoclastogenic differentiation of RAW264.7 cells by heme oxygenase-1. *Cellular Physiology and Biochemistry*.

[37] Wen W., Lin Y., Ti Z. (2019). Antidiabetic, antihyperlipidemic, antioxidant, anti-inflammatory activities of ethanolic seed extract of Annona reticulata L. in streptozotocin induced diabetic rats. *Front Endocrinol*.

[38] Lee S. K., Park D. Y., Lee H. J. (2007). Functional interaction between nitric oxide-induced iron homeostasis and heme oxygenase-1 in immortalized and malignant oral keratinocytes. *Cancer Letters*.

[39] Peng Z., Liao Y., Chen L. (2019). Heme oxygenase-1 attenuates low-dose of deoxynivalenol-induced liver inflammation potentially associating with microbiota. *Toxicology and Applied Pharmacology*.

[40] Liu Y. T., Lin Z. M., He S. J., Zuo J. P. (2019). Heme oxygenase-1 as a potential therapeutic target in rheumatic diseases. *Life Sciences*.

[41] Song L., Li J., Yuan X. (2017). Carbon monoxide-releasing molecule suppresses inflammatory and osteoclastogenic cytokines in nicotine- and lipopolysaccharide-stimulated human periodontal ligament cells via the heme oxygenase-1 pathway. *International Journal of Molecular Medicine*.

[42] Choi E. Y., Choe S. H., Hyeon J. Y., Choi J. I., Choi I. S., Kim S. J. (2015). Carbon monoxide-releasing molecule-3 suppresses Prevotella intermedia lipopolysaccharide-induced production of nitric oxide and interleukin-1*β* in murine macrophages. *European Journal of Pharmacology*.

[43] El Sayed N., Cosgarea R., Rahim S., Giess N., Krisam J., Kim T.-S. (2019). Patient-, tooth-, and dentist-related factors influencing long-term tooth retention after resective therapy in an academic setting-a retrospective study. *Clinical Oral Investigations*.

[44] Zhao D., Khawaja A. T., Jin L. (2020). Effect of non-surgical periodontal therapy on renal function in chronic kidney disease patients with periodontitis: a systematic review and meta-analysis of interventional studies. *Clinical Oral Investigations*.

[45] Vadori M., Seveso M., Besenzon F. (2009). In vitro and in vivo effects of the carbon monoxide-releasing molecule, CORM-3, in the xenogeneic pig-to-primate context. *Xenotransplantation*.

[46] Ollivier A., Foresti R., El Ali Z. (2019). Design and biological evaluation of manganese- and ruthenium-based hybrid CO-RMs (HYCOs). *ChemMedChem*.

